# Ibrutinib repurposing: from B-cell malignancies to solid tumors

**DOI:** 10.18632/oncoscience.310

**Published:** 2016-06-10

**Authors:** Daniel Massó-Vallés, Toni Jauset, Laura Soucek

**Affiliations:** Vall d'Hebron Institute of Oncology (VHIO), Barcelona, Spain; Institució Catalana de Recerca i Estudis Avançats (ICREA), Barcelona, Spain; Department of Biochemistry and Molecular Biology, Universitat Autònoma de Barcelona, Bellaterra, Spain

**Keywords:** ibrutinib, drug repurposing, solid tumors, inflammation, BTK

Ibrutinib (Imbruvica^®^, also known as PCI-32765) is a first-in-class, irreversible small-molecule inhibitor of Bruton's Tyrosine Kinase (BTK) that binds covalently to cysteine C481 within the ATP-binding pocket. Since BTK is a Tec family non-receptor tyrosine kinase that is specifically required for B-cell antigen receptor (BCR) signaling, ibrutinib was initially developed for the treatment of B-cell malignancies. Currently, it is approved for first-line treatment of chronic lymphocytic leukemia, and for the treatment of mantle-cell lymphoma and Waldenström's macroglobulinemia patients that have received at least one previous therapy.

However, BTK is also involved in signaling pathways downstream of many other receptors and its expression is not restricted to B cells. In fact, it is expressed in all hematopoietic lineages with the exception of T lymphocytes. Taking advantage of this aspect of BTK biology, some groups, including ours, have exploited the utility of inhibiting BTK in different cell types other than B cells. In particular, we validated the critical role of BTK for mast cell degranulation in mouse models of pancreatic cancer, namely insulinoma [1] and pancreatic ductal adenocarcinoma (PDAC) [2]. Ibrutinib was effective in both scenarios, leading to vasculature collapse and tumor regression in insulinoma and to a potent and unexpected anti- fibrotic effect in PDAC, where it synergized with standard of care chemotherapy to extend mice survival. This latter observation suggests that the anti- fibrotic activity of ibrutinib could be beneficial also for the treatment of other fibrotic diseases, an aspect that deserves further studies. More recently, Gunderson and colleagues contributed to the understanding of ibrutinib therapeutic impact in PDAC by describing how BTK regulates B-cell and macrophage-mediated T-cell suppression and demonstrating that ibrutinib restores T-cell dependent anti-tumor responses and triggers growth inhibition [3]. These studies combined led to the initiation of a phase 2/3 clinical trial for the use of ibrutinib in combination with nab- paclitaxel and gemcitabine in metastatic PDAC.

Importantly, emerging data obtained from various mouse models of cancer suggests that beneficial use of ibrutinib could extend beyond pancreatic cancer to other solid tumors. For example, myeloid-derived suppressor cells (MDSCs) express BTK and are present in the stroma of many different tumors, causing immunosuppression and evasion of anti-tumor immune responses. Consistently, treatment of breast cancer and melanoma mouse models with ibrutinib reduced the number of MDSCs in the spleen and the tumor, and combination therapy with anti-PD-L1 resulted in reduced mammary tumor growth [4]. Moreover, synergy of ibrutinib with immune checkpoint blockade has also been reported in mouse models of lymphoma, breast and colon cancer, but in a BTK-independent manner [5]. In this case, suppression of tumor growth was due to inhibition of the interleukin-2–inducible T-cell kinase (ITK), an enzyme required by Th2 T cells, allowing a shift of T-cell immune responses to a Th1 bias. However, off-target (BTK-independent) effects of ibrutinib are not limited to ITK, since its inhibitory activity affects several other kinases including Tec, EGFR, HER2, HER3, HER4, JAK3, Blk, Fgr, Hck, Lck, Yes/YES1, Bmx/Etk and Txk. While this lack of selectivity could explain some of its side effects, it could be exploited for the treatment of tumors without BTK dependency. Indeed, two recent reports exemplify this approach by showing promising *in vitro* data against EGFR-mutant or overexpressed non-small cell lung cancer (NSCLC) [6] and HER2-positive breast cancer.

Finally, expression of BTK variants was found in cancer cells of breast, prostate [7] and colon tumors [8]. In all cases, its downregulation by RNAi or its inhibition by pharmacological means, including ibrutinib, leads to impaired proliferation and/or cell death.

All these findings broaden the spectrum of tumor types potentially susceptible to treatment with ibrutinib. Its use against solid tumors, alone or in combination with standard therapies or novel immune-based approaches, is increasingly becoming a clinically viable option. Indeed, a number of clinical trials are already underway for the treatment of different solid tumor types, such as EGFR mutant NSCLC (http://ClinicalTrials.gov Identifier: NCT02321540), refractory stage IV cutaneous melanoma (NCT02581930), metastatic pancreatic adenocarcinoma (NCT02562898 and NCT02436668), localized prostate cancer (NCT02643667), advanced gastrointestinal and genitourinary tumors (NCT02599324), advanced carcinoid and pancreatic neuroendocrine tumors (NCT02575300) and other relapsed or refractory solid tumors including lung and breast (NCT02403271). Results of these trials will shed light on the applicability of ibrutinib in human patients beyond hematological malignancies and quickly open the way to new immediate clinical applications. Nevertheless, further work will be needed in order to establish, for every tumor type, the contribution of BTK-related responses versus inhibition of other kinases, either in tumor cells themselves or in the tumor stroma.

Ibrutinib constitutes a prime example of therapeutic switching, but there are likely many other drugs already in clinical use that are candidates for repositioning, and could offer new, safe and easily-implementable opportunities for cancer patients.
